# Gallbladder hemorrhage during orally administered edoxaban therapy: a case report

**DOI:** 10.1186/s13256-019-2328-9

**Published:** 2019-12-26

**Authors:** Hideya Itagaki, Suzuki Katuhiko

**Affiliations:** Department of General Surgery, Honjou Daiichi Hospital, 110 Iwabuchishita, Yurihonnjou, Akita 015-8567 Japan

**Keywords:** Direct oral anticoagulant (DOAC), Edoxaban, Gallbladder hemorrhage, Hemobilia, Upper gastrointestinal hemorrhage

## Abstract

**Background:**

Edoxaban is an orally administered anticoagulant treatment that is used in patients with cerebral infarction, venous thrombosis, or other conditions, with a reported incidence of gastrointestinal hemorrhage at approximately 1%. We encountered the rare case of a patient who developed a gallbladder hemorrhage after the administration of edoxaban.

**Case presentation:**

An 86-year-old Japanese woman visited our gastrointestinal department due to the chief complaint of melena lasting for a week. Her medical history included hypertension and embolic cerebral infarction, and she was taking orally administered carvedilol (5 mg/day) and edoxaban (30 mg/day). Her palpebral conjunctiva was pale during a physical examination, indicating the possibility of anemia. Her blood test results confirmed severe anemia with red blood cells at 1.7 × 10^6^/μL and hemoglobin at 4.7 g/dL. An upper gastrointestinal endoscopy revealed bile and fresh blood on the duodenal bulb and in more distal regions; hemobilia was suspected. A computed tomography scan on the ninth hospitalization day confirmed the hemobilia with a gallbladder fundus high-density signal. She was discharged on the 30th day of hospitalization with only fluid therapy and no progression of anemia. Moreover, she underwent a laparoscopic cholecystectomy 1 month after discharge, but the pathologist did not identify false aneurysms or neoplastic lesions. She has not been shown to develop anemia for 5 months after surgery.

**Conclusions:**

Our case suggests that gallbladder hemorrhage needs to be considered a possible complication for patients on direct oral anticoagulants.

## Background

Edoxaban is a direct oral anticoagulant (DOAC) directly inhibiting factor Xa [1] which can be used in treating cerebral infarction and venous thromboses caused by non-valvular atrial fibrillation [2]. Hemorrhage is a possible reported adverse effect, but the incidence of gastrointestinal hemorrhage is approximately 1% for patients on high-dose edoxaban (60 mg/day) and warfarin, and the incidence should be lower than 1% in patients on low-dose edoxaban (30 mg/day) [3, 4]. We found no reports of gallbladder bleeding due to edoxaban. However, edoxaban was probably the cause of the gallbladder hemorrhage in our patient.

## Case presentation

**Fig. 1:**
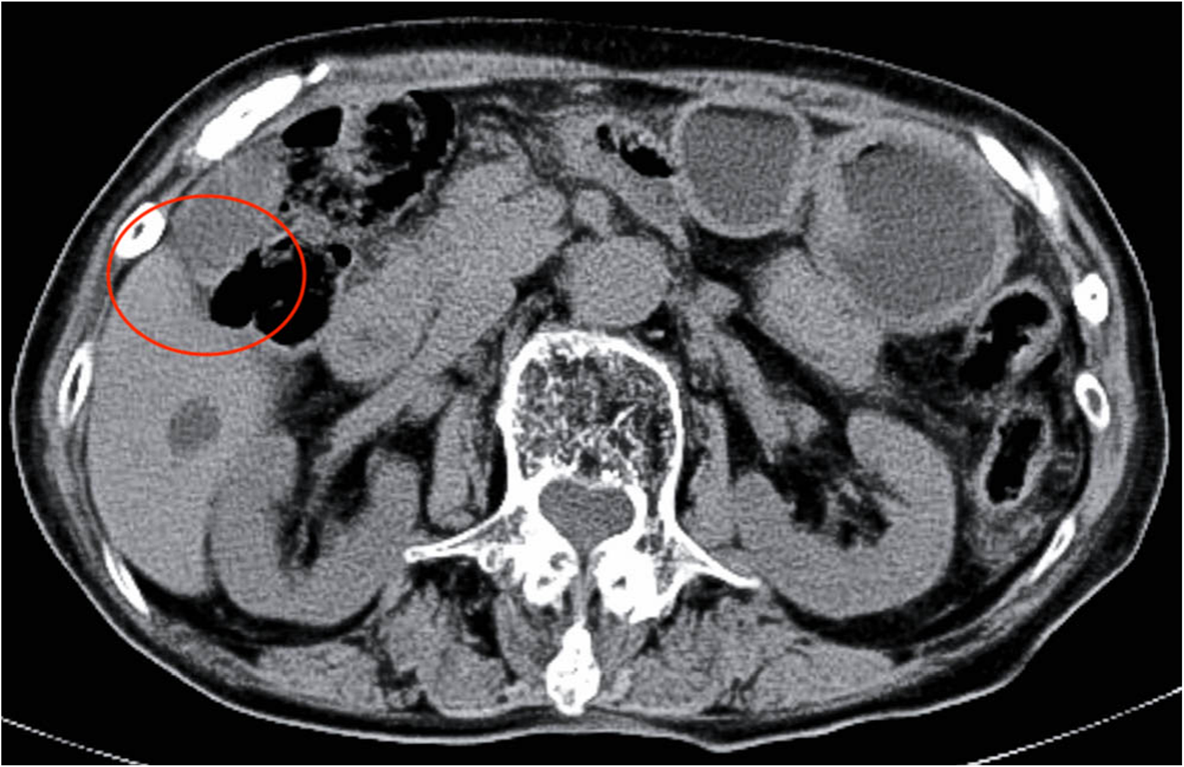
High-density image at gallbladder fundus in a computed tomography scan (*red circle*)

**Fig. 2:**
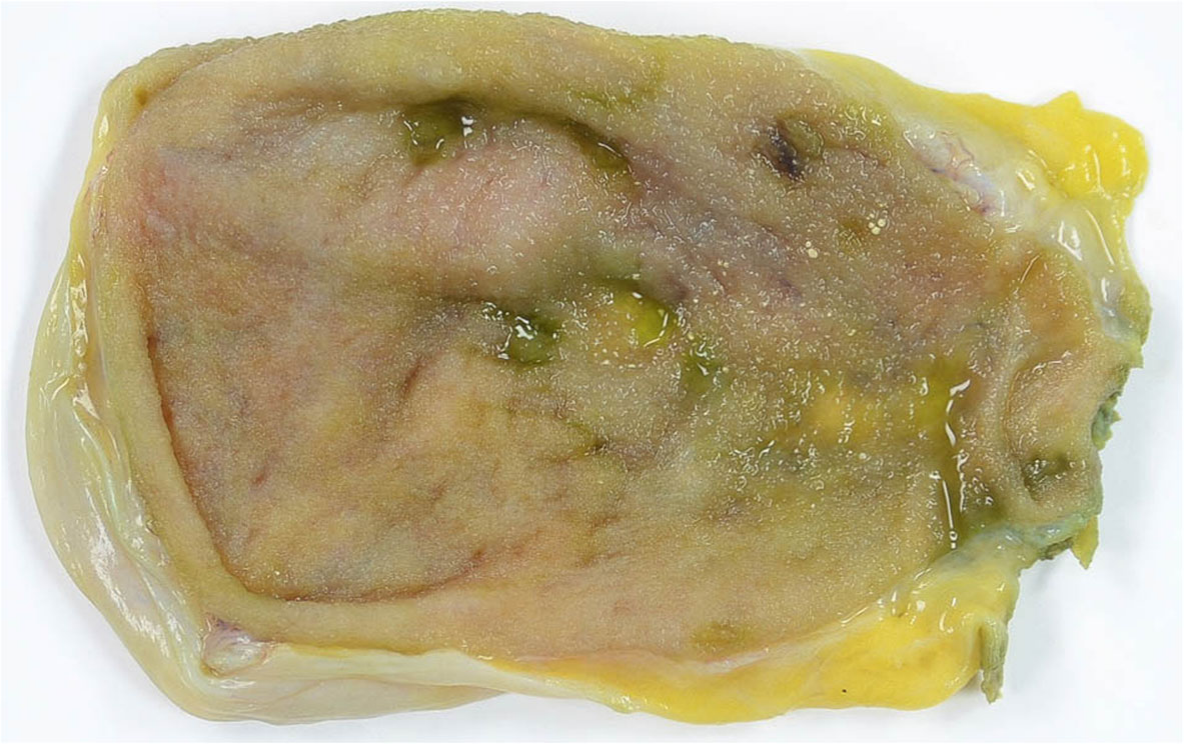
Cholecystectomized gallbladder

**Fig. 3:**
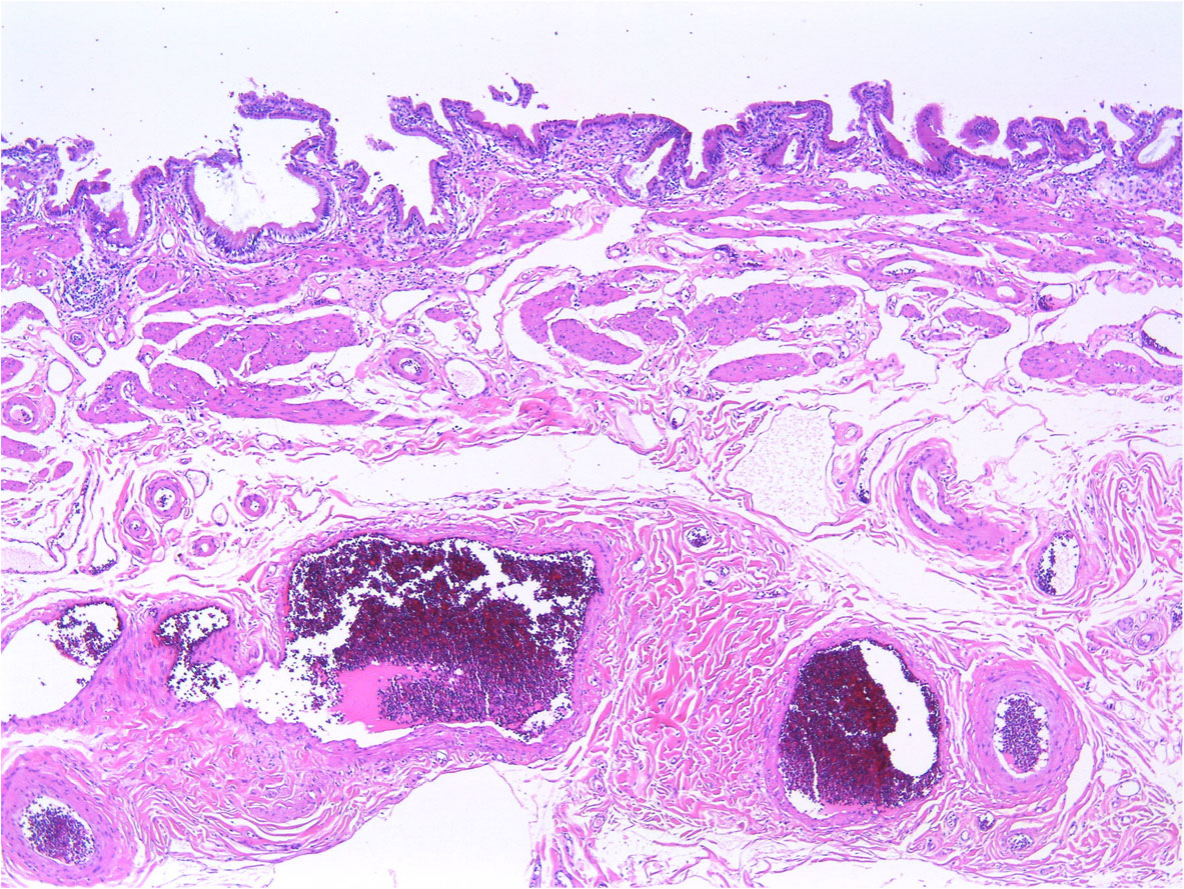
Chronic mild cholecystitis without aneurysm or tumor

An 86-year-old Japanese woman visited the gastrointestinal department at our institution because of the chief complaint of melena that had lasted for a week. She had visited a local doctor and had received a prescription for the H_2_ receptor antagonist Protecadin (lafutidine) but decided to visit our institution after seeing that the melena persisted. She was taking carvedilol (5 mg/day) and edoxaban (30 mg/day) for her preexisting conditions which included hypertension and embolic cerebral infarction. Edoxaban was used for infarction prevention. We did not find hypotension, tachycardia, or abdominal pain during a physical examination upon admission, but the paleness of her palpebral conjunctiva suggested the presence of anemia. Her blood test results indicated severe anemia with red blood cells (RBC) at 1.7 × 10^6^/μL, hemoglobin (Hb) at 4.7 g/dL, and her blood urea nitrogen (BUN)/creatine ratio (52.1 mg/dL versus 1.29 mg/dL) indicated dissociation. Thus, we suspected an upper gastrointestinal hemorrhage and conducted an upper gastrointestinal endoscopy on the following day. We did not find a clear ulcerated lesion during an upper endoscopy; however, hemobilia was suspected due to such findings as bile and fresh blood at the duodenal bulb and more distal regions. We also performed an ultrasound examination on the following day, yielding no clear findings. We transfused our patient for the anemia caused by the hemobilia, and a computed tomography (CT) scan on the ninth hospitalization day due to the persistent Hb reductions revealed a high-density image in her gallbladder fundus (Fig. 1), diagnosed as a gallbladder hemorrhage. We discharged our patient on the 30th hospitalization day after confirming a lack of hemorrhage by upper gastrointestinal endoscopy. Edoxaban was stopped until upper gastrointestinal endoscopy confirmed no bleeding, and resumed after discharge. A magnetic resonance cholangiopancreatography (MRCP) 2 weeks after discharge confirmed the absence of biliary stenosis or pancreaticobiliary maljunction. One month after the discharge, she underwent a laparoscopic cholecystectomy. The pathological findings included mild chronic cholecystitis, without false aneurysms or tumorous lesions (Figs. 2 and 3). For 5 months after surgery, she has not had anemia, and we follow her on an out-patient basis at our institution.

## Discussion

Hemobilia, a rare pathological cause of upper gastrointestinal hemorrhage, was initially reported by Nauyn. Gallbladder hemorrhage is an infrequent cause of hemobilia [5, 6]. Atherosclerotic lesions on the gallbladder wall may lead to hemorrhage [7], but other causes include acute cholecystitis, gallstones, malignant tumors, trauma, hemophilia, aneurysms, or the use of orally administered anticoagulants [6, 8]. Moreover, gallbladder hemorrhage is difficult to distinguish from cholecystitis based solely on physical findings, because they both cause right hypochondriac pain and a positive Murphy sign, and an imaging test may be required to confirm a diagnosis [9]. An echographic examination may reveal a blood clot in the lumen with wall thickening and fluid accumulation around the gallbladder, but these findings are only present in 74% of patients [10]. High-density images in a CT scan on the basis of gallbladder hemorrhage suspicion could be more informative than an echography [11]. For treatment, interventional radiology (IVR) or surgery can be selected depending on the patient’s clinical condition [9]. In this case report, we describe the processes up to the diagnosis of gallbladder hemorrhage and the treatment of our patient. She had visited our institution due to the chief complaint of melena, without abdominal pain. After an unremarkable echographic examination, she was diagnosed as having a gallbladder hemorrhage based on high-density gallbladder images in a CT scan. We chose not to perform emergency IVR or surgery due to the option of treating the anemia by blood transfusions and to avoid surgical complications due to her condition, but we selected laparoscopic cholecystectomy at a later date.

As for the cause of the gallbladder hemorrhage, the pathologist found only chronic inflammation and discarded the presence of acute inflammation, gallstones, malignant tumors, and aneurysms/arteriosclerotic lesions. Moreover, she lacked a prior medical history of trauma before her hospitalization. The coagulation markers measured later during the hospitalization (we did not measure levels initially) showed both normal activated partial thromboplastin time (APTT) and prothrombin time (PT) levels at 29.2 seconds and 10.4 seconds, respectively. The lack of a medical history of intra-articular or intramuscular hematomas ruled out hemophilia. In addition, acute cholecystitis was ruled out based on the lack of A/B/C items in the Tokyo Guidelines 2018 [12]. Item A includes local clinical signs with: (1) a Murphy sign and (2) a palpable mass, spontaneous pain, or right upper quadrant tenderness. Item B includes systemic inflammatory findings like: (1) fever, (2) increased C-reactive protein (CRP) level, and (3) high white cell counts. On the other hand, item C includes images characteristic of acute cholecystitis. The guidelines require all A/B/C items to be present for a diagnosis of acute cholecystitis. In the case of our patient, she denied having abdominal pain, and we found no abdominal tenderness, and she had normal liver enzymes and lacked inflammatory reaction markers. In addition, an echographic examination found no evidence of cholecystitis such as hyperplasia or gallbladder wall expansion. The CT scan on the ninth hospitalization day showed no gallbladder wall hyperplasia or expansion, or neighboring liquid effusions. Thus, we ruled out acute cholecystitis as a cause for the gallbladder hemorrhage. Based on the clinical picture, a gallbladder hemorrhage was suspected induced by an anticoagulant drug (edoxaban), although we are aware that edoxaban is not reported to cause hemorrhages frequently compared to other conventional anticoagulant agents.

Edoxaban is an orally administered anticoagulant drug that directly suppresses factor Xa with linear and predictable pharmacokinetics [1]. According to an international collaborative phase III study called “Effective Anticoagulation with Factor Xa Next Generation in Atrial Fibrillation–Thrombolysis in Myocardial Infarction 48 (ENGAGE AF-TIMI 48)” for patients with atrial fibrillation and a moderate to a high risk of cerebral infarct, the incidences of gastrointestinal hemorrhage were 1.23% for warfarin, 1.51% for high-dose edoxaban, and 0.87% for low-dose edoxaban [4]. Moreover, an open-label trial/non-inferiority trial, based on a comparison between low molecular weight heparin and edoxaban against cancer-related venous thrombosis, showed that the incidence of hemorrhage was 6.9% for edoxaban (significantly higher than the 4% for low molecular weight heparin with a hazard ratio of 1.77) at 60 mg/day or at other high doses [13]. From these trials, low-dose edoxaban may not lead to a significant hemorrhagic tendency compared to other conventional anticoagulant drugs, and it is less likely to cause hemorrhages compared to other anticoagulant drugs. Our patient weighed 48 kg and her body mass index (BMI) was 20. Her creatinine level was 1.29 mg/dL and her estimated creatinine level clearance was 32 ml/minute. So, to prevent the recurrence of embolic stroke, she took edoxaban at a low dose; however, a gallbladder hemorrhage occurred. Conventional anticoagulant drugs (for example, warfarin/heparin) and antiplatelet drugs (for example, aspirin/cilostazol) have been shown to cause gallbladder hemorrhage [8, 14], but only apixaban has been reported to cause gallbladder hemorrhage as a DOAC [9], and we found no reports on cases caused by edoxaban. Moreover, the report on apixaban causing gallbladder hemorrhage was for a patient with gallstone or gangrenous cholecystitis [9], and we think our case lacking gallstones or acute inflammation is worth reporting.

## Conclusion

As the frequency of patients prescribed DOACs increases, we think it is important to consider gallbladder hemorrhage as a possible adverse effect, even in patients who lack acute inflammation or gallstones.

## Data Availability

Data sharing is not applicable to this article as no datasets were generated or analyzed during the current study.

## Abbreviations

CT: Computed tomography
DOAC: Direct oral anticoagulant
Hb: Hemoglobin
IVR: Interventional radiology
